# Recognizing the unconscious

**DOI:** 10.1016/j.cub.2014.09.035

**Published:** 2014-11-03

**Authors:** Trevor T.-J. Chong, Masud Husain, Clive R. Rosenthal

**Affiliations:** 1 Department of Experimental Psychology, University of Oxford, Oxford OX1 3UD, UK; 2 Nuffield Department of Clinical Neurosciences, University of Oxford, Oxford OX3 9DU, UK

## Abstract

Recognition memory enables us to discriminate whether an event has occurred in the past, and is widely interpreted to reflect the conscious retrieval of episodic traces or familiarity 1, 2. Non-conscious mnemonic influences, such as repetition priming, are thought to have a negligible effect on standard tests of recognition memory [3]. A major difficulty with this conclusion is that it is exclusively based on the results from experimental protocols that use stimulus materials available to conscious perception. In eight experiments (*N* = 144), we tested the necessity of mechanisms related to conscious perception for accurate recognition memory by manipulating observers’ awareness of either the encoded event and/or the retrieval cues. Remarkably, observers made accurate objective and subjective recognition memory-guided judgments without visual awareness of the encoded events, retrieval cues or, most strikingly, *both*. These results demonstrate that non-conscious processes can drive accurate recognition memory, and are a significant challenge to neurobiological accounts centered on the conscious retrieval of episodic traces or familiarity.

## Teaser

Chong et al. report evidence that non-conscious processes can drive accurate recognition memory, a significant challenge to neurobiological accounts centered on the conscious retrieval of episodic traces or familiarity.

## Main Text

**Figure 1:**
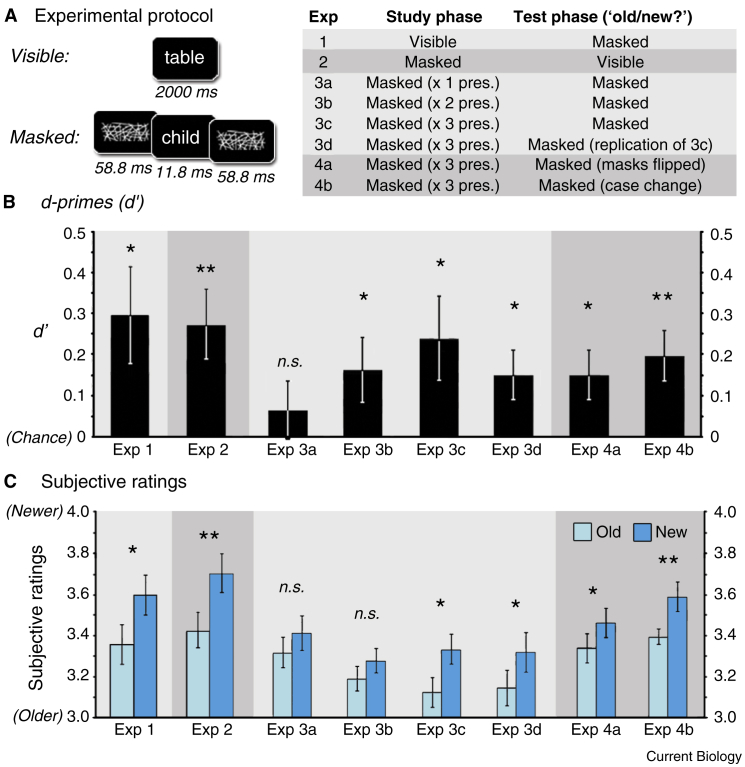
Experimental protocol and results of Experiments 1 to 4b. (A) Word stimuli were either visible (presented for 2000 ms) or masked (presented for 11.8 ms and backward- and forward-masked for 58.8 ms). Each experiment involved a study and test phase, with masked words used in at least one of these phases. At study, observers provided an animacy judgement in response to each word. After a five-minute interval, observers were presented with word-based retrieval cues, and provided an old/new judgement to each word, and then rated the confidence associated with their response. (B) Sensitivity to the difference between old and new word-based retrieval cues was calculated using *d*’ scores (chance = 0). (C) Subjective ratings were based on observers’ confidence associated with each old-new response (from 1, ‘certain old’, to 6, ‘certain new’). ^∗∗^*p* < 0.005; ^∗^*p* < 0.05; *n.s.* not significant.

In Experiment 1, we tested the prediction that *non-conscious retrieval cues* support recognition memory for visible words presented at study (Figure 1A). In the study phase, observers performed trial-wise animacy judgments on 40 *visible words*. After a five-minute interval, word-based retrieval cues (11.8 ms) were presented during a discrete test phase. These retrieval cues were rendered non-conscious by backward and forward masks (58.8 ms), and observers were asked to: (1) decide whether the masked retrieval cue was old or new; (2) rate the confidence associated with each old-new discrimination on a six-point scale; and (3) report the identity of the masked cue. A signal detection theory-based objective measure (*d*’ = 0.30, *t*_(17)_ = 2.74, *p* < 0.01; Figure 1B) and subjective confidence ratings (*t*_(17)_ = 2.87, *p <* 0.01; Figure 1C) revealed accurate discrimination between masked old and new retrieval cues. Importantly, this occurred despite the inability of observers to report the identity of the retrieval cues (accuracy = 2%).

In Experiment 2, we asked the reciprocal question: can words masked from visual awareness *at study* support accurate recognition with visible retrieval cues? To assess whether the words were adequately masked, observers rated the confidence of animacy judgements on a three-point scale. The results revealed that, even though masking led to chance performance on animacy judgments at study (50.7%, *t*_(17)_ = 0.4, *n.s.*), significant recognition memory was evident on the objective (*d*’ = 0.27, *t*_(17 )_= 3.32, *p* < 0.005) and subjective (*t*_(17)_ = 3.24, *p* < 0.005) measures. Evidence from Experiments 1 and 2 is consistent with a view that conscious and non-conscious based mnemonic mechanisms can interact in service of accurate recognition memory.

In six additional experiments, words were masked from visual awareness at *both* study *and* test to identify whether non-conscious memory alone can drive accurate recognition memory. In Experiment 3a, single presentations of masked words led to chance recognition performance (*d*’ = 0.06, *t*_(17)_ = 0.95, *n.s.*; subjective ratings, *t*_(17)_ = 1.51, *n.s.*). Therefore, we modified the study protocol based on prior evidence that spaced repetition increases the likelihood that an event will be perceived as previously encountered [4]. In Experiment 3b, each masked word was now presented *twice* at study, with all other aspects of the design unchanged from Experiment 3a. Critically, this manipulation led to accurate objective non-conscious discrimination (*d*’ = 0.15, *t*_(17)_ = 2.03, *p* < 0.05), but did not support accurate subjective judgments (*t*_(17)_ = 1.39, *n.s.*).

To establish if repetition could lead to accurate subjective judgments, masked words were next presented *three times* at study (Experiment 3c). Significant non-conscious recognition memory was evident again on the objective measure of discriminability (*d*’ = 0.22, *t*_(17)_ = 2.40, *p* < 0.05), and this was now accompanied by accurate recognition memory in the subjective ratings (*t*_(17)_ = 2.57, *p* < 0.01). We replicated these results in a further experiment (3d) that employed an additional assay of masking adequacy at retrieval. Specifically, trial-wise animacy judgements were performed after each subjective rating, and observers were excluded if animacy judgements between study and test improved (>5%). Despite this additional stringent criterion, significant recognition memory was evident on the objective (*d*’ = 0.14, *t*_(17)_ = 2.47, *p* < 0.05) and subjective measures (*t*_(17)_ = 2.81, *p* < 0.01).

Finally, we examined whether stimulus-specific processing provided a source of fluency that could be used to infer prior occurrence 3, 5. In the first follow-up experiment, we mirror-reversed the masks between study and test (Experiment 4a), and in a second experiment, we changed the word case between study and test from lower-case to upper-case (Experiment 4b). The results replicated the non-conscious recognition effect observed in Experiments 3b–d, and also demonstrated that the effect was not dependent on stimulus-specific fluency, suggesting a possible role for lexical information (Experiment 4a: *d*’ = 0.12, *t*_(17)_ = 2.81, *p* < 0.01; subjective ratings, *t*_(17)_ = 2.83, *p* < 0.01; Experiment 4b: *d*’ = 0.18, *t*_(17)_ = 3.22, *p* < 0.005; subjective ratings, *t*_(17)_ = 3.51, *p* < 0.005).

Conscious memory is unlikely to explain the non-conscious recognition memory effects in Experiments 3–4. This view is supported by three independent sources of evidence. First, semantic encoding is strongly associated with conscious memory, but could not be easily deployed due to visual masking — animacy judgments at study (range 50.6–52.4%, all *t*_(17)_ < 1.67, *n.s.*) and test (Experiment 3d, 50.1 ± 1.1%, *t*_(17)_ = 0.12, *n.s.*) were at chance. Furthermore, encoding accuracy was not correlated with above-chance significant *d*’s in Experiments 2–4b (each *p* > 0.18). Second, the results are based on the removal of studied words associated with accurate animacy judgments and a high confidence rating. Third, there was no difference in the reportability of 20 masked words before and after each experiment, which excludes a perceptual learning-mediated change in the identification threshold (mean difference –0.2–0.6%; all *t*_(17)_ < 1.45, *n.s.*).

In summary, we found evidence of a robust and unprecedented non-conscious recognition memory effect by modifying a conventional recognition memory task protocol. Observers made accurate objective and subjective recognition memory-guided judgments, without conscious access to the words at study and test (Experiments 3–4). These experiments contrast with other paradigms, such as those based on the mere exposure effect, in which incidental encoding modulates affective judgements [6] (for a discussion, see Supplemental Information). The experimental evidence has three immediate implications. First, on the grounds that awareness is widely regarded as a prerequisite for establishing an episodic- or familiarity-driven record or token of an event [7], the results provide an empirical basis on which to generate new hypotheses about the neural signals that can drive recognition memory 8, 9, 10. Second, the protocol enables the effects of recognition-guided retrieval to be distinguished from downstream ‘conscious mechanisms’, because masking excluded processes related to top-down attention; the intention to retrieve prior episodes; perceptual expectations; and the recovery of episodic information. Third, our protocol demonstrates that precluding visual awareness isolates non-conscious components of recognition memory, alongside other techniques based on encouraging guessing in response to highly similar retrieval cues [10]. Applying a protocol that is preferentially sensitive to detecting mnemonic mechanisms operating outside of conscious perception can potentially clarify other controversies, such as the extent to which non-conscious memory mediates hippocampal-dependent tasks [9].

## Supplemental Information

Document S1. Experimental Procedures, Results and Two Tables

## References

[1] Squire L. (2009). Memory and brain systems: 1969–2009. J. Neurosci..

[2] Yonelinas A.P. (2002). The nature of recollection and familiarity: A review of 30 years of research. J. Mem. Lang..

[3] Conroy M.A., Hopkins R.O., Squire L.R. (2005). On the contribution of perceptual fluency and priming to recognition memory. Cogn. Affect. Behav. Neurosci..

[4] Greene R. (1990). Spacing effects on implicit memory tests. J. Exp. Psychol. Learn. Mem. Cogn..

[5] Jacoby L., Dallas M. (1981). On the relationship between autobiographical memory and perceptual learning. J. Exp. Psychol. Gen..

[6] Seamon J., Brody N., Kauff D. (1983). Affective discrimination of stimuli that are not recognized: Effects of shadowing, masking, and cerebral laterality. J. Exp. Psychol. Learn. Mem. Cogn..

[7] Moscovitch M., Gazzaniga M. (1995). The Cognitive Neurosciences.

[8] Dew I., Cabeza R. (2011). The porous boundaries between explicit and implicit memory: behavioral and neural evidence. Ann. NY Acad. Sci..

[9] Henke K. (2010). A model for memory systems based on processing modes rather than consciousness. Nat. Rev. Neurosci..

[10] Voss J.L., Paller K.A. (2009). An electrophysiological signature of unconscious recognition memory. Nat. Neurosci..

