# Supplementary material for: Recognizing the unconscious
Source: Curr Biol. 2014 Nov 3;24(21):R1033–5. doi: 10.1016/j.cub.2014.09.035 (PMC4228049; doi:10.1016/j.cub.2014.09.035)
Supplement: Document S1. Experimental Procedures, Results and Two Tables [file mmc1.pdf]

Supplemental Information: Recognizing the Unconscious

Trevor T-J Chong, Masud Husain, & Clive R Rosenthal

Supplemental Experimental Procedures

Participants

Each experiment comprised separate groups of 18 healthy volunteers (excluding those who did not meet criteria for adequate masking, as explained below). Volunteers were recruited from a university participant panel, with the approval of the local ethics committee. The final sample of 144 volunteers (52% male) had an age range of 18-54 years (mean 26 years).

Materials and Stimuli

For each experiment, we selected 80 common nouns from a standard database (the MRC Psycholinguistic Database) [S1]. Half the words referred to inanimate items (e.g., 'table', 'guitar'), and the other half was animate (e.g., 'horse', 'lawyer'). Each word was five- or six-letters in length, with an average word frequency of 35.8 occurrences per million [S2]. Words presented during the study phases of each experiment had comparable word frequencies and identical average word-lengths to those presented at test (5.5 letters). Words were presented in lower-case, white, Arial 40 point font against a black background at the centre of a cathode ray tube monitor, configured to a refresh rate of 85 Hz. The forward and backward masks comprised hatches of white lines, and the identical mask was used for all trials in this experiment, with the exception of the test phase of Experiment 3d, in which the masks were horizontally mirror-reversed. The viewing distance was 60 cm, and ambient lighting was dimmed. The order of presentation of words and retrieval cues was randomised for each observer.

Preliminary Test of Visual Masking

At the beginning of each session, the efficacy of visual masking for each observer was assessed by examining the ability to identify individual words. Observers were presented with 20 individual masked words, with the instruction to read aloud any words that could be seen. Words used on this identification test were five- or six-letters in length, and were drawn from a separate corpus to that used for the main experiments. Each trial started with a 1000 ms fixation point, followed by a word stimulus lasting 11.8 ms, which was backward- and forward-pattern masked for 58.8 ms. These masking conditions were identical to those used in the main experiments. The experimenter recorded verbal responses to each masked word, and any observer who read more than two of the twenty words correctly (10%) in this preliminary session was excluded (total number of excluded

participants for Experiments 1-4b: 6, 4, 4, 8, 6, 4, 5, 4, 4, respectively). Our chosen parameters therefore represented the identification threshold for each observer. Although this criterion may be more liberal than an awareness or recognition threshold [S3], we address this issue in subsequent re-analyses by removing masked words that were associated with a correct animacy judgement and a high degree of confidence. Furthermore, in additional analyses, we also demonstrate that there was no significant change in the identification threshold over the course of each experiment, by comparing initial performance with an identical post-retrieval session test that probed the efficacy of visual masking (see below).

Experimental Tasks

Each experiment involved two stages: an initial study phase and a subsequent test phase. Across eight experiments, we manipulated whether masked words were presented at study (Experiment 1), test (Experiment 2), or both study and test (Experiments 3a to 3d; Experiments 4a and 4b).

<u>Experiment 1</u>

In Experiment 1, 40 visible words (50% animate; 50% inanimate) were presented at the study phase, and 80 masked words at the test phase. The masking procedures were identical to those described above. The presentation of each visible word at study was followed (after a 1000 ms delay) by a prompt requiring observers to judge the animacy of the target word (animate vs inanimate). Previous studies have shown that depth of processing facilitates the ability to (consciously) recollect an event at study [S4]. These responses were registered with a keyboard, and the inter-trial duration was 2000 ms.

The test phase began five minutes after completion of the study phase. Observers were presented with 80 masked words, half of which were old (i.e., had been presented during the preceding study phase), and the other half new (50% animate; 50% inanimate). Observers were not informed about the distribution of word stimuli in the experiment. Each trial involved the presentation of a masked word-based retrieval cue. Trials started with a 1000 ms fixation point, followed by the onset of mask-word-mask triad. Log files for every observer in every experiment were checked to ensure that there were no lags or dropped buffers in the presentation of the word or mask stimuli. At the conclusion of each trial, observers were instructed to: (1) decide whether the masked retrieval cue was old or new; (2) rate the confidence associated with each old-new discrimination on a six-point scale; and (3) verbally report the identity of the masked cue. If no words were perceived, observers

initiated the next trial by pressing the space bar. All responses were recorded on a keyboard, and the inter-trial duration was 2000 ms.

Experiment 2

Experiment 2 was identical to Experiment 1, except that words in the study phase were masked from visual awareness, and the retrieval cues were visible. In order to ensure that the words in the study phase were adequately masked, observers were also required to provide a confidence rating on a three-point scale, after performance an animacy judgement on each word (1 = most confident; 3 = least confident).

Experiments 3a – 3d

In Experiments 3a–3d, word stimuli were masked at both the study and test phases. Across this series of four experiments, we titrated the number of times observers were exposed to each of the 40 masked words in the study phase. This was based on evidence that spaced repetition increases the likelihood that an event will be perceived as previously encountered [S5]. In Experiment 3a, each word was presented only once; in Experiment 3b, each word was presented twice for a total of 80 trials; and in Experiments 3c–d, each word was presented three times for a total of 120 trials. In Experiments 3b–d, the trial order was organised in a 'massed repetition' design, such that the 40 words were divided into ten blocks of four words each. Each block was then presented consecutively two (Experiment 3b) or three (Experiments 3c–d) times. The order of words within each block of four trials was identical across repetitions of each block. Observers were naïve to this design. Experiment 3d differed from Experiment 3c by additionally requiring observers to perform an animacy judgement after each subjective rating during the test phase. This was performed as an additional test to determine whether the non-conscious recognition memory effects that we observed in Experiments 3c remained intact, when even more stringent masking criteria were applied. In particular, we excluded observers whose animacy judgements improved by more than 5% between study and test (four participants).

Experiments 4a – 4b

Experiments 4a and b investigated the role of stimulus-specific fluency based effects on the novel recognition memory effects that we observed in experiments 3c and 3d. Experiments 4a and b were, therefore, very similar in design to Experiment 3c, where the basic effect on objective and subjective

measures of recognition was demonstrated, with differences only in the stimulus-specific properties of the mask (experiment 4a) or words (experiment 4b) used at test.

Experiment 4a asked whether fluency associated with the mask-word-mask triad had an impact on the non-conscious recognition memory effect seen in Experiments 3c and d. This experiment was similar to Experiment 3c, except that the forward and backward masks used for the test phase were horizontally mirror-reversed relative to their orientation in the study phase. This served two, related purposes: first, it excluded the intrusion of partial orthographic informational overlap between study and test as a possible explanation for the observed non-conscious recognition effect; and, second, it reduced the overall perceptual similarity of the mask-word-mask triad between study and test. This manipulation therefore allowed us to alter the perceptual representations of the mask-word-mask triad between study and test, and thereby reduce the possibility that fluency associated with the mask-word-mask triad generated a signal that could be used to infer its prior occurrence [S6, S7, S8].

Experiment 4b also tackled the issue of whether the non-conscious recognition memory effects in Experiment 3c were driven by stimulus-specific fluency related to the re-presented word. The design of this final experiment was similar to that of Experiment 3c, with the critical manipulation being that the words presented at test were presented in upper-case letters, in contrast to the words at study which were presented in lower-case. The cap height (i.e., the height from the base to the top) of the upper-case letters was equal to the x-height (i.e., the height of a lowercase ‘x') of the lower-case letters.

Assessment of perceptual learning over the course of each experiment

The role of perceptual learning was assessed by re-presenting observers with the list of 20 masked words in the same way as in the preliminary test of masking. Observers were again asked to read aloud any words that could be seen, and these data were compared against performance on the first test of masking from visual awareness.

Supplemental Results

Data from all experiments are summarised in Table S1. *p* values for *d′* measurements and old/new subjective judgements are reported as one-tailed tests.

Experiment 1 – Visible words at study; Masked retrieval cues

As predicted, animacy judgements on the unmasked words at study were at ceiling (mean = 98.9%). In analyzing data from the test phase, we eliminated words that were correctly identified during the study phase. Overall, the mean proportion of retrieval cues that were correctly identified by verbal report was 2%, and we derived corrected signal detection and subjective rating measures by removing these intrusions from our analyses.

Standard signal detection analyses were performed on data from the test phase in order to calculate observers' sensitivity (*d′*) and response bias (β) (Supplementary Table S1). *d′* was therefore calculated as the difference between the normalized hit and false alarm rates (*Z(H) – Z(FA)*), and β (natural log) was calculated as $-d' \times 0.5 \times (Z(H) + Z(FA))$. These analyses revealed that *d′* that was significantly above chance ($d' = 0.30$, $t_{(17)} = 2.74$, $p < .01$), with no evidence of response bias ($\beta = 0.10$, $t_{(17)} = 1.20$, *n.s.*).

Finally, we compared observers' subjective ratings associated with the old and new retrieval cues. We recoded observers' responses onto a six-point scale (from 1 = Certain Old to 6 = Certain New). Analyses of their subjective responses showed that they consistently rated veridically old items as older than veridically new items (old 3.31 vs new 3.61, $t_{(17)} = 2.87$, $p < .01$).

Experiment 2 – Masked words at study; Visible retrieval cues

The average accuracy for animacy judgements in the study phase was 50.7%, which was not significantly different from chance ($t_{(17)} = 1.67$, *n.s.*). The majority of confidence ratings during the study phase were categorised as '2' (50.8%) or '3' (36.3%), with observers responding '1' on relatively few trials (12.4%). These data were used to eliminate the effect at the test phase of masked words that were better perceived during the study phase. For each observer, we identified the words during the study phase that were associated with a correct animacy judgement and which had a high subjective rating (i.e., a rating of '1'). We then derived corrected measures of *d′* and β by excluding these words from the subsequent signal detection analysis. Despite this correction, the *d′s* remained significantly above chance (mean *d′* .27, $t_{(17)} = 3.32$, $p < .005$), and there was no shift in

response bias (β = 0.05, $t_{(17)}$ = 1.70, *n.s.*). Importantly, there was no correlation between the mean accuracy of animacy judgments during the study phase and the *d'* scores (r = -0.11, p = .66). Furthermore, there was a significant difference between observers' subjective ratings associated with old as compared with new items (old 3.42 vs new 3.71, $t_{(17)}$ = 3.24, $p < .005$).

Experiment 3a – Masked words at study with single word presentations; Masked retrieval cues

Mean accuracy of animacy judgments at study were not significantly different from chance (51.1%, $t_{(17)}$ = 0.78, *n.s.*). The corrected *d'*s did not reach significance (*d'* = 0.06, $t_{(17)}$ = 0.95, *n.s*), and there was no evidence of a significant response bias (β = 0.00, $t_{(17)}$ = 0.20, *n.s.*). Subjective ratings for old and new items were also not significantly different from chance (old 3.40 vs new 3.52, $t_{(17)}$ = 1.51, *n.s.*).

Experiment 3b – Masked words at study with double word presentations; Masked retrieval cues

Mean accuracy of animacy judgments at study remained at chance (52.4%, $t_{(17)}$ = 1.62, *n.s.*). However, the two presentations of each word at study led to a significant above chance *d'* value (*d'* = 0.15, $t_{(17)}$ = 2.03, $p < .05$). There was again no evidence of a response bias (β = 0.00, $t_{(17)}$ = 0.15, *n.s.*). Importantly, there was no correlation between encoding accuracy and *d'* (r = 0.08, p = .75). Subjective ratings for veridically old and new items were not significantly different (old 3.24 vs new 3.35, $t_{(17)}$ = 1.39, *n.s.*).

Experiment 3c – Masked words at study with triple word presentations; Masked retrieval cues

Mean accuracy of animacy judgments at study was at chance, as in experiment 3b (51.6%, $t_{(17)}$ = 1.67, *n.s.*), thereby indicating that target words were masked from visual awareness. Here, three presentations of each target word supported accurate recognition memory (*d'* = 0.22, $t_{(17)}$ = 2.40, $p < .05$). Response biases were not significantly different from zero (β = -0.03, $t_{(17)}$ = 0.53, *n.s.*), and the correlation of encoding accuracy with *d'* was not significant (r = 0.31, p = .21). Subjective ratings for veridically old and new items were significantly different (old 3.15 vs new 3.42, $t_{(17)}$ = 2.57, $p < .01$).

Experiment 3d – Masked words at study with triple word presentations; Masked retrieval cues with additional trial-wise animacy judgements.

The mean accuracy for animacy judgements in the study phase was 51.6% $\pm$ 0.9%, which was not significantly different from chance ($t_{(17)}$ = 1.73, *n.s.*). Critically, overall animacy judgements during the test phase were also not significantly different from chance (50.1 $\pm$ 1.1%, $t_{(17)}$= 0.12, *n.s.*), nor were they different to those at study ($t_{(17)}$ = 1.35, *n.s.*). Observers' *d'* values were significantly different from chance (*d'* = 0.14, $t_{(17)}$= 2.47, $p < .05$), and there was no significant evidence of a criterion shift (β = 0.00, $t_{(17)}$ = 0.06, *n.s.*). Furthermore, there was no significant correlation between *d'* scores and animacy accuracy at either study (r = 0.29, p = .25) or test (r = 0.33, p = .18). As in Experiment 3c, evidence of significant recognition memory in *d'* was accompanied by a significant difference in the subjective confidence ratings between old and new retrieval cues (old 3.19 vs new 3.41, $t_{(17)}$ = 2.81; $p < .01$). Overall, therefore, this experiment replicates Experiments 3c, and shows that above-chance non-conscious recognition memory remains demonstrable with masking criteria that are even more stringent than those used in the preceding experiments. Moreover, the data also demonstrate that masked encoding with triple word presentations did not have a detectable impact on visual awareness associated with the studied words, as assessed on a stringent set of objective criteria that included animacy judgments at test as well as at study.

Experiment 4a – Masked words at study with triple word presentations; Masked retrieval cues with mirror-reversed masks

In the same way as in previous experiments, target words were successfully masked from visual awareness on the basis that performance on the animacy judgment task was at chance (51.7%, $t_{(17)}$ = 1.19, *n.s.*). Importantly, we replicated the results from Experiment 3c after reversing the mask in the test phase. *d'* was significantly greater than chance (*d'* = 0.12, $t_{(17)}$ = 2.81, $p < .01$). There was no significant response bias (β = 0.02, $t_{(17)}$ = 0.92, *n.s.*), and no significant correlation between *d'* and the accuracy of the animacy judgment (r = 0.31, p = .22). Subjective ratings were again significantly different between old and new retrieval cues (old 3.43 vs new 3.59, $t_{(17)}$ = 2.83, $p < .01$). This implies that the accurate recognition memory performance observed in Experiment 3c was not dependent on stimulus-specific fluency related to the re-presentation of the same mask-word-mask triad.

Experiment 4b – Masked words at study with triple word presentations in lower-case; Masked retrieval cues with words in upper-case

The mean accuracy of animacy judgements was 51.7%, which was not significantly different from chance ($t_{(17)}$ = 1.53, *n.s.*). Critically, the mean *d'* during the test phase was significantly greater than chance (*d'* 0.18, $t_{(17)}$ = 3.22, $p < .005$), in the absence of a shift in criterion (β= 0.04, $t_{(17)}$ = 1.84, *n.s.*).

Furthermore, significant recognition memory on the objective measure was accompanied by a significant difference in the subjective ratings between veridically old and new items (old 3.51 vs new 3.75, $t_{(17)} = 3.51$, $p < .005$). In agreement with all of the preceding experiments, there was no correlation between encoding accuracy and *d'* ($r = 0.03$, $p = .91$).

Together, Experiments 3a-d and 4a-b represent a significant departure from previous studies on recognition memory by presenting non-conscious words at *both* study and test. Previous work on the mere exposure effect has shown that repeated exposure to a subliminally-encoded stimulus increases affective judgements of liking that stimulus, despite participants being at chance in recognising whether that item had been previously presented [S9, S10, S11].

In contrast, observers in our study were above-chance in recognising the subliminally-encoded stimuli in Experiments 2 and 3a-d. One potential reason for this apparent discrepancy may be related to differences in strategy and task requirements. Studies of the mere exposure effect often rely on incidental encoding, in contrast to the present experiments that oriented observers to semantic features of the masked stimuli. Observers in mere exposure effect based tasks may therefore rely more heavily on non-conscious fluency, in contrast to observers in our task whose intentional orientation to the masked words may have facilitated their above-chance performance at test. Indeed, previous studies have shown that participants can be encouraged to use levels of processing fluency in order to infer the prior occurrence of a stimulus (c.f. fluency attribution [S12]; analytic/holistic strategies [S13]). Thus, an important implication to arise from this study is that the strategy which observers use to process a non-conscious signal can be critical in determining the outcome.

Tests of Perceptual Learning

We conducted several analyses to ensure that perceptual learning could not account for the non-conscious recognition memory effects in experiments 3b-d and experiments 4a-b. First, of the corpus of 20 words presented during the preliminary test of visual masking, the mean percentage that was correctly identified was across all of the experiments was 2.74% ($\pm$ SEM 0.4%). This was not significantly different from the percentage of correctly identified words when the test of perceptual learning was repeated at the end of the recognition test (2.7% $\pm$ SEM 0.4%, $t_{(143)} = 1$, *n.s.*).

Second, if perceptual learning occurred over time, we would expect observer performance during the study phase of each experiment to improve with time, as observers learn the low-level properties of the mask. We tested for this possibility by examining the accuracy of animacy judgements during the first and second halves of each study phase (see Table S2). Critically, in seven

of the eight experiments, there were no significant differences in the accuracy of animacy judgements between the first and second halves of the study phase. In Experiment 3b, performance actually appeared to decline between the first and second halves. It is, therefore, possible to exclude the possibility that any habituation to the mask over time was relevant to the observed non-conscious recognition memory effect.

Third, in Experiment 3d, one would predict perceptual learning to manifest as improvements in animacy judgements from study to test. However, no such significant differences were found, because the accuracy of animacy judgements was the same between study and test (study 51.6 $\pm$ 0.9% vs test 50.1 $\pm$ 1.1%; $t_{(17)} = 1.35$, *n.s.*).

Finally, if above-chance recognition in the test phase simply reflected memory for the mask-word-mask triad, one would have expected that changing the stimulus-specific properties of the mask during the test phase should have had an impact on recognition memory. However, the results of Experiment 4a, in which we mirror-reversed the mask at test, showed that the novel non-conscious recognition memory effect was significant, in spite of this manipulation. Overall, therefore, there was no evidence to support that perceptual learning was responsible for the robust evidence of significant recognition memory without visual awareness.

Supplementary Analyses with Unequal Variance Signal Detection Methods

One possible reason to explain why animacy judgements were at chance, whereas objective and subjective recognition memory based judgements were above-chance, may be that old words in the test phase had, by definition, already been previously presented. This raises the issue of how old and new items are distributed. Indeed, much memory research (on supraliminal stimuli) suggests that the variance of the distribution of old items may be greater than that of new items [S14].

Standard signal detection analyses assume equal variance between the distribution of old and new items. In an additional analysis, we therefore estimated observers' *d′* using an unequal variance signal detection model [S15], which can be expressed as:

$$p(Y \leq k \mid X) = \Phi \frac{c_k - d_n X}{\sigma_s^X}$$

for $k = 1$ to $K - 1$, where K is the number of response categories (here 6), Y is a response variable that takes on values $k = 1$ to K (in this case, the confidence ratings from 1 to 6); X is a dummy coded variable (here, old = 0; new = 1); $p(Y \leq k \mid X)$ is the cumulative probability of a response of k or less given X; Φ is the cumulative normal distribution function; c_k are response criteria (distances from

the distribution of new items) with the property $c_1 < c_2 < \ldots < c_{K\text{-}1}$; d_n is the detection parameter scaled with respect to the standard deviation of the distribution of new items (σ_n, which is set to unity); and σ_s is the standard deviation of the distribution of old items (i.e., the signal distribution).

The results of applying an unequal variance signal detection model to our data are summarised in Table S2. Essentially, the detection parameters derived from this model (d_n) yielded the same pattern of results across all experiments, with the exception of Experiment 3b (two presentations of masked words at study, with masked retrieval cues), in which the unequal variance detection parameter fell just outside significance (d' = 0.08; $t_{(17)}$ = 1.56; p = .068). However, analyses of all subsequent iterations of the masked study and test experimental protocol (Experiments 3c-d, 4a-b) using an unequal variance model revealed significant and commensurate detection parameters when compared to the standard analyses that were based on equal variance.

Table S1

Summary of encoding accuracy and signal detection analyses for all experiments. Values are given for detection parameters derived from equal variance (d') and unequal variance signal detection models (d_n). Bold values indicate significant results (p < .05).

Experiment	Accuracy of animacy judgement	*d'*	d_n	Rating Old vs New
1	98.9% [a]	**0.30 (*p* = .007)**	**0.12 (*p* = .03)**	**3.31 vs. 3.61 (*p* = .005)**
2	50.7%	**0.27 (*p* = .002)**	**0.19 (*p* = .02)**	**3.42 vs. 3.71 (*p* = .002)**
3a	51.1%	0.06 (*p* = .18)	0.07 (*p* = .13)	3.40 vs. 3.52 (*p* = 0.07)
3b	52.4%	**0.15 (*p* = .03)**	0.12 (*p* = .07)	3.24 vs. 3.35 (*p* = 0.09)
3c	51.6%	**0.22 (*p* = .01)**	**0.19 (*p* = .02)**	**3.15 vs. 3.42 (*p* = .01)**
3d	51.6% [b], 50.1% [c]	**0.14 (*p* = .01)**	**0.18 (*p* = .007)**	**3.19 vs. 0.41 (*p* = 0.006)**
4a	51.7%	**0.12 (*p* = .006)**	**0.17 (*p* = .004)**	**3.43 vs. 3.59 (*p* = .006)**
4b	51.5%	**0.18 (*p* = .003)**	**0.17 (*p* = .002)**	**3.51 vs. 3.75 (*p* = .001)**

[a] *Words visible at study in Experiment 1*

[b] *Accuracy of animacy judgements at study*

[c] *Accuracy of animacy judgements at test*

Table S2

Percentage accuracy of animacy judgements during the first and second halves of the study phase for masked trials. There were no significant improvements in encoding accuracy between the first and second halves of each study phase. Accuracy is given as mean (SEM) percentages. Statistics are provided as t-values (with corresponding p-values).

	**Exp.2**	**Exp.3a**	**Exp.3b**	**Exp.3c**	**Exp.3d**	**Exp.4a**	**Exp.4b**
**1st Half**	51.1 (2.7)	51.9 (1.5)	55.4 (2.0)	50.8 (1.6)	50.5 (1.7)	52.8 (1.7)	51.8 (1.5)
**2nd Half**	50.4 (2.6)	50.3 (2.2)	49.6 (2.0)	52.4 (1.8)	52.8 (1.8)	50.6 (1.8)	51.6 (1.6)
t-value	0.13	0.67	-2.03	0.94	0.80	1.05	0.08
(p-value)	(.45)	(.26)	(.03)	(.18)	(.22)	(.16)	(.47)
			N.B. 1st>2nd				

Supplemental References

S1. Coltheart, M. (1981). The MRC Psycholinguistic Database. Quarterly Journal of Experimental Psychology *33A*, 497-505.

S2. Kucera, H., and Francis, W.N. (1967). Computational analysis of present-day American English, (Providence: Brown University Press).

S3. Reingold, E., and Merikle, P. (1988). Using direct and indirect measures to study perception without awareness. Perception and Psychophysics *44*, 563-575.

S4. Roediger, H.L., and McDermott, K.B. (1993). In Handbook of Neuropsychology, F. Boller and J. Grafman, eds. (Amsterdam: Elsevier).

S5. Greene, R. (1990). Spacing effects on implicit memory tests. Journal of Experimental Psychology: Learning, Memory and Cognition *16*, 1004-1011.

S6. Berry, C., Shanks, D., Speekenbrink, M., and Henson, R. (2012). Models of recognition, repetition priming, and fluency: exploring a new framework. Psychological Review *119*, 40-79.

S7. Conroy, M.A., Hopkins, R.O., and Squire, L.R. (2005). On the contribution of perceptual fluency and priming to recognition memory. Cognitive, Affective, and Behavioral Neuroscience *5*, 14-20.

S8. Jacoby, L., and Dallas, M. (1981). On the relationship between autobiographical memory and perceptual learning. Journal of Experimental Psychology: General *110*, 306-340.

S9. Seamon, J., Marsh, R., and Brody, N. (1984). Critical importance of exposure duration for affective discrimination of stimuli that are not recognized. Journal of Experimental Psychology: Learning, Memory and Cognition *10*, 465-469.

S10. Zajonc, R. (2001). Mere exposure: A gateway to the subliminal. Current Directions in Psychological Science *10*, 224-228.

S11. Seamon, J., Brody, N., and Kauff, D. (1983). Affective discrimination of stimuli that are not recognized: Effects of shadowing, masking, and cerebral laterality. Journal of Experimental Psychology: Learning, Memory and Cognition *9*, 344-355.

S12. Jacoby, L., Kelley, C., and Dywan, J. (1989). Memory attributions. In Varieties of Memory and Consciousness: Essays in Honour of Endel Tulving, H.I. Roediger and F. Craik, eds. (Hillsdale, NJ: Lawrence Erlbaum), pp. 391-422.

S13. Whittlesea, B., and Price, J. (2001). Implicit/explicit memory versus analytic/nonanalytic processing: Rethinking the mere exposure effect. Memory and Cognition *29*, 234-246.

S14. Wixted, J. (2007). Dual-process theory and signal-detection theory of recognition memory. Psychological Review *114*, 152-176.

S15. Green, D., and Swets, J. (1966). Signal Detection Theory and Psychophysics, (New York: Wiley).
